# P-2019. Improving Biomedical Waste Segregation Through the Implementation of a QR Code–Enabled Mobile Audit and Alert System: A Quality Improvement Study in a Tertiary Care Hospital

**DOI:** 10.1093/ofid/ofaf695.2183

**Published:** 2026-01-11

**Authors:** Jomel Raju, Maria Tom, Leena James

**Affiliations:** St. Joseph's College of Pharmacy, Cherthala, Pala, Kerala, India; St. Joseph's College of Pharmacy, Cherthala, Pala, Kerala, India; St. Joseph's College of Pharmacy, Elampally, Kerala, India

## Abstract

**Background:**

Improper biomedical waste (BMW) segregation is a critical patient and staff safety concern in healthcare institutions, particularly in resource-limited settings. Traditional monitoring methods using manual checklists are often delayed, nonstandardized, and ineffective in triggering timely corrective actions. This quality improvement study aimed to assess the impact of a QR code–enabled mobile audit and alert system on improving biomedical waste segregation compliance in a tertiary care hospital in South India.

**Figure:**
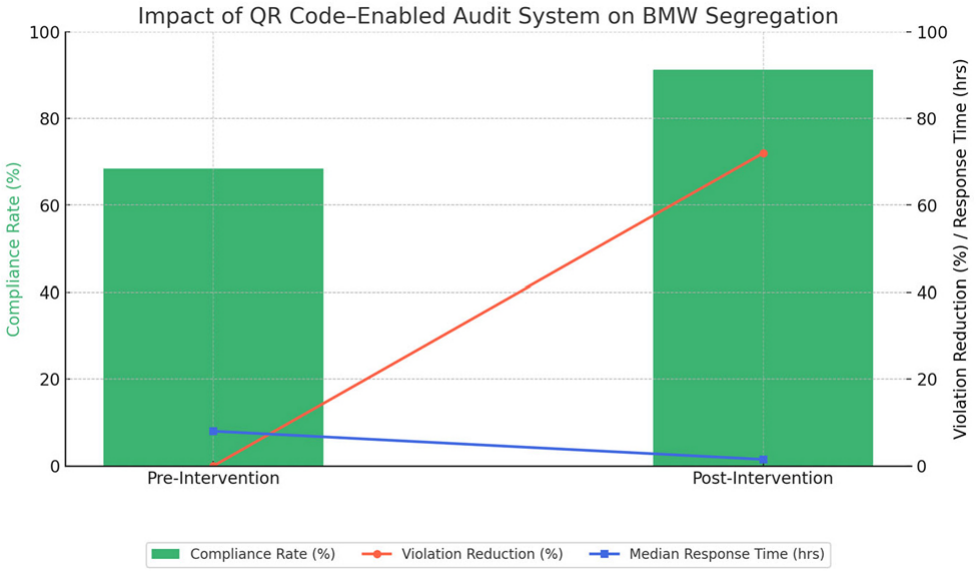
Impact of QR Code–Enabled Audit System on Biomedical Waste Segregation Compliance and Response Metrics

The green bars represent an improvement in biomedical waste segregation compliance from 68.4% to 91.2% over the 12-week post-intervention period.

The red line shows a 72% reduction in category mismatch violations after the intervention.

The blue line indicates a reduction in median response time to noncompliance alerts, decreasing from 8 hours to 1.5 hours.

Together, these findings highlight the system's effectiveness in strengthening real-time monitoring and timely corrective actions in resource-limited settings.

**Methods:**

A prospective interventional study was conducted from January to June 2024 in a 650-bed tertiary care teaching hospital. A custom mobile application with QR codes installed at waste disposal points in 12 high-risk areas (ICUs, operation theaters, and emergency units) was used to conduct daily BMW segregation audits. Noncompliance triggered automated alerts to the Infection Control Nurse and Unit Supervisor in real time. Pre-intervention data were collected over 4 weeks using traditional paper checklists. Pre- and post-intervention compliance rates were compared using the chi-square test, and user feedback was assessed through the System Usability Scale (SUS).

**Results:**

Baseline BMW segregation compliance was 68.4%. Following implementation, compliance improved to 91.2% over 12 weeks (p < 0.001). The number of category mismatch violations reduced by 72%. Median response time to noncompliance alerts decreased from 8 hours to 1.5 hours. The mobile system was rated highly usable with a mean SUS score of 82.7. Over 85% of auditors reported increased ease of documentation and monitoring.

**Conclusion:**

The QR code–enabled audit and alert system significantly improved BMW segregation compliance, reduced response delays, and enhanced real-time monitoring. This model offers a scalable and cost-effective digital solution for improving infection control and waste management practices in similar healthcare settings.

**Disclosures:**

All Authors: No reported disclosures

